# Multi-ancestry genome-wide association study of serum creatine kinase implicates myopathy genes and muscle pathways

**DOI:** 10.1016/j.ebiom.2026.106274

**Published:** 2026-04-30

**Authors:** Gang Chen, Sizheng S. Zhao, Hector Chinoy, James B. Lilleker, Weijie Liu, Yuhui Li, Andrew P. Morris, Janine A. Lamb

**Affiliations:** a Epidemiology and Public Health Group, School of Health Sciences, The University of Manchester, Manchester, UK; b Centre for Musculoskeletal Research, Division of Musculoskeletal and Dermatological Sciences, Faculty of Biology, Medicine and Health, The University of Manchester, Manchester, UK; c NIHR Manchester Biomedical Research Centre, Manchester University NHS Foundation Trust, Manchester Academic Health Science Centre, Manchester, UK; d Department of Rheumatology, Salford Royal Hospital, Northern Care Alliance NHS Foundation Trust, Manchester Academic Health Science Centre, Salford, UK; e Manchester Centre for Clinical Neuroscience, Manchester Academic Health Science Centre, Salford Royal Hospital, Northern Care Alliance NHS Foundation Trust, Salford, UK; f Department of Rheumatology and Immunology and Beijing Key Laboratory for Rheumatism and Immune Diagnosis, Peking University People's Hospital, Beijing, China

**Keywords:** Creatine kinase, Genome-wide association study, Multi-ancestry, Skeletal muscle

## Abstract

**Background:**

Serum creatine kinase (CK) is a routinely measured biomarker of muscle damage, yet the genetic factors underlying inter-individual variation in CK levels remain poorly defined.

**Methods:**

Here we present a large multi-ancestry genome-wide association meta-analysis of serum CK, comprising 237,255 participants spanning Admixed American, African American, East Asian, European and Middle Eastern populations.

**Findings:**

We identify 107 independent loci at genome-wide significance (*P*< 5 × 10^−8^), 98 of which are previously unreported, with pronounced enrichment for genes expressed in skeletal and cardiac muscle and overlap with pathways related to muscle structure and function. Notably, eight loci map to genes implicated in Mendelian myopathies, underscoring a continuum from common regulatory variation to rare pathogenic mutations. Integrative quantitative trait locus (QTL)-based Mendelian randomisation and colocalisation implicate several genes in CK regulation, most prominently *SMAD3*, *KLF5* and *STAT3* within the transforming growth factor beta signalling pathway. CK levels show positive genetic correlations with traits reflecting tissue damage as well as muscle mass and strength, and negative correlations with C-reactive protein, indicating pleiotropic effects from muscle biology and enzyme clearance.

**Interpretation:**

These findings delineate the genetic architecture of serum CK across diverse populations and highlight muscle-related pathways contributing to CK variation.

**Funding:**

No funding was received for this study.

**Research in context**

**Evidence before this study**

Serum creatine kinase (CK) is routinely used to detect muscle damage, but normal ranges vary widely by sex, ancestry, muscle mass, and physical activity, complicating distinction between physiological and pathological elevations. Previous genome-wide association studies, mainly in Europeans, identified a few loci (including *LILRB5*, *ANO5*, *CKM*), linking CK to statin-associated myopathy and adult-onset muscular dystrophy and suggesting that mild CK elevation can reflect subclinical muscle disease. However, the broader genetic architecture of CK across ancestries, its systematic overlap with Mendelian myopathy genes, and its relation to molecular markers of tissue damage and inflammation have not been comprehensively characterised.

**Added value of this study**

In a multi-ancestry GWAS meta-analysis of 237,255 individuals, we identified 107 CK-associated loci (98 previously unreported), with strong enrichment for genes expressed in skeletal and cardiac muscle and for genes causing Mendelian muscle disorders. Eight lead-variant genes overlapped a curated Mendelian muscle-disease set (odds ratio 14), and many exonic variants lay in classic myopathy genes (*TTN*, *NEB*, *ANO5*, *CACNA1S*, *MYPN*, *CAV3*), consistent with a continuum from rare, high-impact mutations to common alleles that highlight biological pathways related to muscle integrity and CK leakage. Functional prioritisation with eQTL and pQTL Mendelian randomisation and colocalisation highlighted regulators of TGF-β signalling (*SMAD3*, *KLF5*, *STAT3*) and immune pathways (*LILRB5*, *GCKR*), providing biological context for how common variation may influence muscle structure, repair, and CK clearance. By focussing on population biobank data, the study primarily captures genetic determinants of subclinical CK variation and muscle-related determinants of CK release/clearance in population biobank settings.

**Implications of all the available evidence**

Taken together, available evidence indicates that inter-individual differences in CK partly reflect inherited variation in muscle mass, sarcolemmal stability, and repair, as well as immune-mediated enzyme clearance. CK-raising alleles in Mendelian myopathy genes probably act as modifiers, contributing to inter-individual differences in muscle integrity and CK leakage that could become clinically relevant with triggers such as intense exercise, infection, or statin therapy. In this context, persistent idiopathic CK elevation may warrant closer follow-up evaluation in appropriate clinical contexts. Strong genetic correlations and shared causal variants with AST, ALT, creatinine, and muscle traits show that modest CK and AST elevations often have a muscular rather than hepatic origin, whereas some findings are consistent with a contribution of immune-mediated clearance to CK levels. These findings motivate future work to evaluate whether incorporating polygenic and Mendelian information can help refine reference ranges, avoid unnecessary investigation of benign elevations, and identify individuals in whom even mild CK rises warrant closer monitoring.

## Introduction

Creatine kinase (CK) is a key enzyme that catalyses the adenosine triphosphate (ATP)-dependent phosphorylation of creatine, thereby sustaining energy homoeostasis in tissues with high and fluctuating metabolic demands, notably skeletal muscle, myocardium and brain.^1^ Elevated serum CK is a well-established biomarker of tissue damage, observed following strenuous or unaccustomed exercise as well as in conditions ranging from benign myalgia and statin-associated myopathy to severe rhabdomyolysis.^1^^,^^2^ Accordingly, CK measurement is routinely employed in exercise physiology and sports medicine to monitor exercise-induced muscle injury and in the clinical assessment of muscle pathology. However, serum CK concentrations exhibit substantial inter-individual variability, influenced by factors such as age, sex, genetic ancestry, physical activity, and muscle mass.^3^ Baseline levels are typically higher in males than in females, and individuals of Black African ancestry often present with markedly elevated CK relative to other ancestry groups.^3^ This heterogeneity complicates the interpretation of CK as a biomarker, as variation in baseline concentrations may hinder the distinction between physiological increases and pathological elevations.

Genome-wide association studies (GWAS) in individuals of European ancestry have identified multiple genetic loci associated with serum CK levels, including *ANO5*, *CD163*, and *LILRB5*.^4^^,^^5^ The largest study to date, comprising 63,159 Icelandic individuals, incorporated stratification by statin use to investigate CK variation.^4^ One of the strongest associations was observed for a common missense variant in *LILRB5*. Further studies implicated this *LILRB5* variant in increased susceptibility to statin intolerance and myalgia.^6^ Variants in *ANO5*, a gene associated with adult-onset muscular dystrophies,^7^ also demonstrated modest associations with elevated CK levels, suggesting a continuum of genetic influences spanning subclinical biochemical alterations to myopathic disease. These findings reinforce the utility of CK as a sensitive biomarker of subclinical muscle damage and point to its potential in revealing the molecular basis of mild, often unrecognised, muscle pathologies in the general population.

Large-scale biobanks have catalysed major advances in GWAS of circulating biomarkers by integrating genomic data with detailed clinical and phenotypic information. Analyses of circulating biomarkers, including C-reactive protein (CRP) and troponin, in cohorts such as UK Biobank and INTERVAL have identified genetic loci linked to inflammation and cardiac injury, providing insights into disease pathophysiology.^8^^,^^9^ More recent GWAS in extensively phenotyped cohorts—such as the Million Veteran Program (MVP) and BioBank Japan (BBJ)—have leveraged electronic health records (EHRs) and standardised laboratory assays to map loci influencing serum CK levels across diverse ancestries.^10^^,^^11^ Despite these advances, most studies have prioritised statistical associations over functional annotation or mechanistic interpretation. Furthermore, reliance on single biobank datasets limits the resolution of causal inference and reduces the generalisability of findings across populations.

In this context, we conducted a large multi-ancestry GWAS meta-analysis of serum CK levels, including 237,255 individuals from diverse populations. Our analyses aimed to define the genetic architecture of CK, identify biological pathways contributing to its variation, and provide insight into muscle-related biology as well as determinants of CK release and clearance. These findings advance understanding of the population-level biology of serum CK and provide a foundation for future studies of its clinical interpretation.

## Methods

### Ethics

All data analysed in this work were de-identified summary statistics obtained from Vanderbilt University Medical Centre (VUMC), MVP, BBJ, and Qatar Genome Program (QGP), each collected under prior ethics approval and participant consent. No individual-level data were accessed, and no new ethical approval was required for this secondary analysis.

### Cohorts, phenotypes, and genotyping

We performed a meta-analysis using six cohorts of ancestry-specific GWAS summary statistics from four biobanks: MVP^11^ (African, Admixed American, European ancestries), BBJ^10^ (East Asian ancestry), VUMC^12^ (European ancestry) and QGP^13^ (Middle Eastern ancestry), encompassing a total of 237,255 individuals. GWAS summary statistics were provided by each biobank and were generated from their respective data resources using established cohort-specific protocols (Supplementary Table S1).

Cohort-defined CK phenotypes were derived from routine clinical laboratory testing (MVP, VUMC/BioVU and BBJ) or diagnostic laboratories at Hamad General Hospital during Qatar Biobank assessments (QGP). Phenotypes were processed within each cohort using established pipelines, including record/unit cleaning, outlier filtering and within-cohort standardisation. Repeated CK measurements were summarised per participant using the mean in MVP and the median in VUMC/BioVU, whereas BBJ and QGP analysed CK as a baseline biomarker. CK was analysed on a cohort-standardised scale using rank-based inverse normal transformation in QGP, MVP and VUMC/BioVU, and log-transformation followed by Z-score standardisation in BBJ. Cohort-specific phenotype processing details are provided in Supplementary Table S1.

Genotypes were generated using array-based platforms with imputation in MVP, BBJ and VUMC/BioVU, and whole-genome sequencing in QGP, with cohort-specific variant and sample QC.^10^, ^11^, ^12^, ^13^ In the source GWAS, covariates and software used in the original cohort association testing pipelines are shown in Supplementary Table S1.

### Quality control and data harmonisation

Per-cohort GWAS summary statistics were quality-controlled using the EasyQC^14^ pipeline supplemented with custom scripts. At the cohort level, we harmonised input formats, excluded rare variants (minor allele frequency <1%), structural variants, and single nucleotide polymorphisms (SNPs) with missing or non-AC or GT alleles. SNP identifiers were standardised across cohorts, and all variants were mapped to the GRCh37 (hg19) reference genome. At the meta-analysis level, we evaluated data integrity and potential biases using a suite of diagnostic tools, including SE–N and P–Z plots, allele frequency concordance checks, and genomic inflation statistics.

### Lead variant concordance across cohorts

To evaluate consistency of genetic associations between cohorts, we compared effect estimates of lead variants across studies. Genome-wide significant variants (*P* < 5 × 10^−8^) were identified independently within each cohort and combined into a candidate lead variant list. To define independent signals, variants were clumped using linkage disequilibrium (LD) estimates from the 1000 Genomes Project^15^ (all populations) with a 1 megabase (Mb) window and an R^2^ threshold of 0.1. Pairwise concordance of effect directions and magnitudes was assessed by calculating Pearson's correlation coefficients between effect sizes across cohorts.

### Meta-analysis of genome-wide association statistics

We conducted meta-analyses using the fixed-effect inverse-variance weighted (IVW) approach and the Stouffer's method as implemented in METAL.^16^ For the European ancestry meta-analysis, which included the MVP European ancestry and VUMC cohorts, we applied the IVW method. For the multi-ancestry meta-analysis, IVW was used when the BBJ cohort was excluded, whereas the Stouffer method was applied when BBJ was included, to account for differences in data transformation and effect size of BBJ. The largest multi-ancestry meta-analysis, including BBJ, was used for locus discovery, whereas the European-only meta-analysis was used for functional follow-up. The multi-ancestry analysis excluding BBJ provided complementary estimates of effect sizes and enabled assessment of between-cohort heterogeneity using Cochran's Q, the corresponding heterogeneity *P* value (P_het), and I^2^ as implemented in METAL.^16^ Quantile–quantile (QQ) plots and genomic inflation factors (λ) were generated to evaluate residual test statistic inflation. Variants located within the human leucocyte antigen region (chromosome 6: 25–34 Mb, hg19) were excluded from all analyses. All other variants were retained for subsequent locus discovery and functional annotation.

### Gene- and gene-set enrichment analysis

Gene- and gene-set association analyses were performed using Multi-marker Analysis of GenoMic Annotation (MAGMA)^17^ with summary statistics from the European GWAS meta-analysis. SNPs were mapped to protein-coding genes, and gene–level association statistics were calculated by aggregating SNP-wise effects while accounting for LD using the 1000 Genomes Project European reference panel, as implemented in Functional Mapping and Annotation (FUMA).^18^ Gene-level *P* values were subsequently used to assess enrichment of predefined gene sets, including tissue- and cell type-specific expression profiles from the Genotype-Tissue Expression (GTEx)^19^ and the Human Protein Atlas,^20^ as well as curated pathway sets available through FUMA.^18^ Multiple testing correction for both gene- and gene-set analyses was applied using Bonferroni adjustment.

### Locus discovery and functional annotation

Genome-wide significant loci were identified using the largest multi-ancestry meta-analysis dataset. For each chromosome, the variant with the smallest *P* value was designated as the lead SNP. A ±500 kilobases (kb) window was applied around each lead SNP, and all variants within this interval were assigned to the same locus. This procedure was repeated iteratively until no additional variants surpassed the genome-wide significance threshold (*P* < 5 × 10^−8^). To distinguish unreported from previously reported associations, we curated a reference list of known variants from the prior CK-related GWAS.^4^^,^^5^^,^^21^ Previously unreported loci were defined as those without a previously reported association within ±1 Mb of the lead SNP.

Functional annotation of lead variants was performed using ANNOVAR,^22^ mapping to genes in the UCSC refGene sequences (release 2020-08-22) and assessing pathogenicity information through ClinVar^23^ (version 2024-09-17). Previously reported associations were retrieved from the GWAS Catalogue,^24^ and regulatory effects were evaluated by integrating skeletal muscle expression quantitative trait loci (eQTL) from GTEx v8.^19^

### Enrichment in muscle Mendelian disease gene set

Genes associated with Mendelian disorders were obtained from the OMIM database^25^ and restricted to phenotypes with a confirmed molecular basis (phenotype mapping key = 3). The gene set was curated to include muscle-specific disorders by JBL. CK-associated loci were mapped to genes using UCSC refGene annotations, and intergenic loci were excluded. The primary analysis considered genes corresponding to lead SNPs. As a sensitivity analysis, we broadened the CK-associated gene set to include genes mapped from all genome-wide significant SNPs (*P*< 5 × 10^−8^) in the multi-ancestry meta-analysis (including BBJ), rather than restricting to lead variants. Only protein-coding genes with an approved symbol from the HUGO Gene Nomenclature Committee were retained, defining a background of 17,554 genes. Enrichment of CK-associated genes within the Mendelian muscle disease gene set was evaluated using a 2 × 2 contingency table, and statistical significance was assessed with Fisher's exact test (two-sided; *P*< 0.05).

### Gene prioritisation with Mendelian randomisation, colocalisation, and exercise transcriptomics

We assessed causal relationships between gene expression and serum CK using molecular quantitative trait loci (QTL) summary based Mendelian randomisation (SMR) with the HEIDI test^26^ on European-ancestry GWAS summary statistics. Analyses were conducted with the SMR portal, which integrates pre-computed QTL data. CK GWAS summary statistics were integrated with eQTL from GTEx v8 across relevant tissues (skeletal muscle, whole blood, heart atrial appendage, heart left ventricle, liver, visceral adipose, and subcutaneous adipose)^19^ and circulating protein quantitative trait locus (pQTL) from the Fenland study.^27^ Associations were considered significant at *P* < 0.05 after Bonferroni correction; HEIDI *P* > 0.05 was interpreted as consistent with vertical pleiotropy rather than linkage.

Colocalisation analyses were subsequently performed for 107 loci identified in the multi-ancestry meta-analysis (including BBJ) using the ‘coloc’ R package.^28^ For each locus, colocalisation was assessed within ±500 kb of the lead variant, comparing European-ancestry GWAS effects with GTEx eQTL data^19^ from corresponding tissues.

We queried MetaMEx^29^ to evaluate whether genes prioritised by CK GWAS–muscle eQTL colocalisation show differential expression in human skeletal muscle in response to exercise or inactivity. Meta-analysed log fold-change (logFC) estimates and 95% confidence intervals were extracted for acute aerobic exercise, acute high-intensity interval training (HIIT), acute resistance exercise, inactivity/unloading, and exercise training interventions (aerobic, HIIT, resistance, and combined). Significance was defined using MetaMEx^29^ multiple-testing–adjusted *P* values (adjusted *P*< 0.05). Results are summarised as forest–style plots stratified by intervention type.

### Heritability and genetic correlation estimation

SNP-based heritability was estimated separately in BBJ, VUMC, the MVP, and a European meta-analysis using LD Score Regression (LDSC)^30^ with ancestry-matched pre-computed LD scores from the 1000 Genomes Project Phase 3 reference panels.

Genetic correlations between serum CK and a comprehensive set of systemic and cardiac phenotypes were quantified using LDSC. The analysed phenotypes comprised two broad categories (Supplementary Tables S13, S14). The first category considered systemic phenotypes^24^^,^^31^ that included circulating biomarkers and clinical traits spanning liver function (e.g., AST, ALT, alkaline phosphatase), renal function (creatinine, cystatin C), lipid and metabolic traits (LDL, HDL, triglycerides, apolipoproteins), inflammatory and haematological indices (C-reactive protein, white and red blood cell counts), musculoskeletal traits (hand grip strength, fat-free mass, height), as well as cardiometabolic and neuromuscular diseases (coronary artery disease, heart failure, type 2 diabetes, amyotrophic lateral sclerosis), among others. The second category included cardiorespiratory fitness^32^ and cardiac structural^33^ and functional^34^ phenotypes, including VO_2_max, physical activity, and imaging-derived measures of left and right ventricular volumes, ejection fraction, stroke volume, myocardial wall thickness, and hypertrophic cardiomyopathy. The European summary statistics for these traits were curated from the GWAS Catalogue^24^^,^^32^, ^33^, ^34^ and OpenGWAS.^31^ To reduce bias from poorly imputed variants, analyses were restricted to high-quality variants across imputation panels, following LDSC best-practice recommendations.^30^

To further explore potential causal relationships between CK and genetically correlated systemic phenotypes, we conducted two-sample Mendelian randomisation (MR) analyses.^35^ Genome-wide significant variants associated with each exposure were selected as instruments and harmonised with outcome summary statistics. CK was assessed bidirectionally as both exposure and outcome. Causal estimates were evaluated using IVW as the primary method. Heterogeneity and directional pleiotropy were assessed using Cochran's Q statistic and MR-Egger intercept,^36^ respectively.

### Trait-based clustering of CK-associated loci

We clustered loci according to their effect sizes across systemic traits related to CK. Effect estimates for CK were obtained from our European GWAS meta-analysis. Eight lead SNPs were excluded owing to limited coverage (reported <30 traits out of 42 traits) and traits with weak genetic correlation to CK ($P_{LDSC}$> 0.05) were also excluded. All effect sizes were aligned to the CK-increasing allele. For each locus–trait pair, we computed sample size adjusted z-scores as Zij=βij(Nisij), where $\beta_{ij}$ denotes the effect estimate, $s_{ij}$ its standard error, and $N_{i}$ the corresponding sample size. Missing values were imputed using the ‘ClustImpute’ R package,^37^ which implements iterative k-means clustering while incorporating phenotypic correlations. The optimal number of clusters was determined with the ‘NbClust’ package,^38^ based on consensus across 27 clustering indices. Associations between traits and cluster membership were assessed by linear regression. Heat maps were generated to visualise the clustering structure, with colour intensity reflecting both the direction of effect and the −log_10_-transformed *P* values. Values were truncated at ± 20 for visualisation.

### Multi-trait colocalisation of CK-associated loci

We applied Hypothesis Prioritisation for multi-trait Colocalisation (HyPrColoc),^39^ a deterministic Bayesian framework designed to identify shared causal variants across systemic traits demonstrating genetic correlation with CK. For each locus, we considered a ±500 kb window centred on the lead variant identified in the multi-ancestry meta-analysis (including BBJ), using effect estimates from the European-specific meta-analysis. Allelic orientations were harmonised across traits to ensure consistency. HyPrColoc was executed with default parameters, incorporating effect sizes and their standard errors. Colocalisation was considered significant when the posterior probability exceeded 0.8.

### Role of funders

There was no study-specific funding source for this work. Individual authors received salary or fellowship support, but these funders had no role in study design, data collection, data analysis, data interpretation, or writing of the report.

## Results

### Analytical framework and cohort concordance

**Fig. 1:**
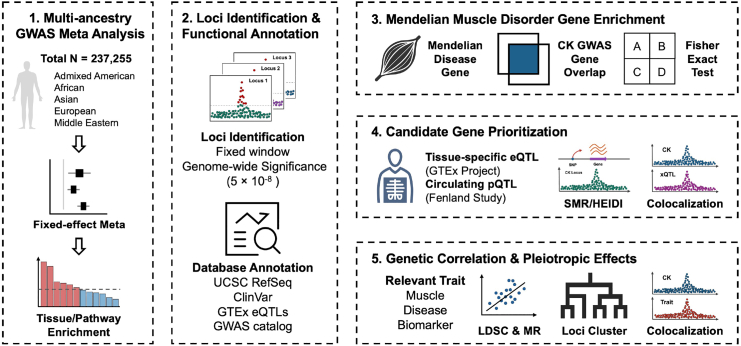
**Overview of multi-ancestry GWAS and functional analyses of serum CK associated loci.** We performed a fixed-effects meta-analysis of GWAS summary statistics for CK levels from six cohorts across diverse ancestries. Associated loci were annotated and tested for enrichment in Mendelian muscle disorder genes. Candidate genes were prioritised using eQTL and pQTL data. Shared genetic architecture with related traits was explored through LD score regression, Mendelian randomisation, and colocalisation. Loci were further clustered according to pleiotropic profiles.

We curated GWAS summary statistics for serum CK levels from six ancestry-specific cohorts from four biobanks: MVP,^11^ BBJ,^10^ VUMC,^12^ and QGP,^13^ comprising a total of 237,255 individuals (Supplementary Table S1). To identify genetic loci associated with serum CK and explore their functional relevance, we implemented a multi-step analytical framework integrating GWAS meta-analysis, functional annotation, enrichment analyses for Mendelian muscle disease genes, candidate gene prioritisation, and genetic correlation (Fig. 1).

Standardised quality control procedures were applied uniformly across all datasets (Methods; Supplementary Figures S1, S2). To assess the consistency of CK associations across cohorts, we compared effect sizes for independent lead variants and observed moderate to high correlations across most ancestry groups (Supplementary Figure S3). The highest pairwise concordance was observed between the European ancestry subset of MVP and VUMC (Pearson's correlation coefficient (r) = 0.859, *P* = 2.94 × 10^−20^), whereas the lowest concordance was noted between VUMC and the African American ancestry subset of MVP cohort (r = 0.141, *P* = 0.297).

### Multi-ancestry meta-analysis and significant muscle enrichment

**Fig. 2:**
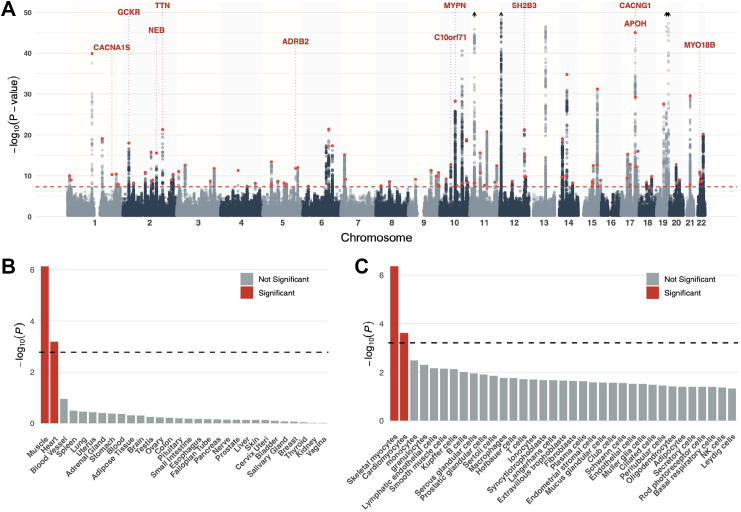
**Multi-ancestry genome-wide association and tissue/cell-type enrichment analyses.** (A) Manhattan plot of multi-ancestry GWAS results for CK across all autosomes; the red dashed line denotes the genome-wide significance threshold (P = 5 × 10^−8^). Previously unreported lead SNPs are shown in red; for clarity only previously unreported loci with exonic lead SNPs are annotated with mapped genes. The y axis is capped at −log_10_(P) = 50; peaks exceeding this value are truncated and marked with arrows, with lead variants and mapped genes rs7305678 (CD163/APOBEC1), rs11559024 (CKM), rs12975366 (LILRB5) and rs7481951 (ANO5). (B & C) MAGMA tissue and cell-type enrichment analysis based on (B) GTEx expression data, (C) Human Protein Atlas single-cell expression profiles; the dashed line indicates the Bonferroni-corrected significance threshold, and significant tissues and cell types are shown in red.

We conducted a large multi-ancestry GWAS meta-analysis of serum CK, leveraging cross-population LD to improve fine-mapping and statistical power. Using Stouffer's method to account for different trait transformations across biobanks, the primary meta-analysis encompassed 17,355,739 SNPs across 237,255 individuals and showed modest genomic inflation (λ = 1.13; Supplementary Table S2), consistent with a polygenic signal rather than uncontrolled confounding, as supported by QQ plots demonstrating deviation confined to the tail of the distribution (Supplementary Figure S4). The Manhattan plot of the multi-ancestry meta-analysis is shown in Fig. 2A. Additional multi-ancestry meta-analyses of biobanks using the same trait transformation (i.e. excluding BBJ), as well as a European ancestry-specific meta-analysis, were performed using an IVW approach (Supplementary Table S2 and Supplementary Figure S5). SNP-based heritability estimates from LDSC were similar across European ancestry (VUMC and MVP European) and East Asian ancestry (BBJ) cohorts, at approximately 0.10 (Supplementary Table S3).

Due to differences in LD structure and the availability of well-powered, ancestry-matched reference panels for gene-based and QTL analyses, downstream functional analyses were based on the European ancestry-specific meta-analysis, which accounted for 38% of the total sample size. Gene- and gene-set enrichment analyses were conducted using MAGMA mapping SNP associations to 18,649 protein-coding genes. Ninety-nine genes surpassed the Bonferroni-corrected significance threshold (*P*≤ 2.69 × 10^−6^; Supplementary Table S4), with *CKM* (*P* = 5.55 × 10^−17^), *WDR73* (*P* = 1.61 × 10^−15^), and *LILRB2* (*P* = 3.94 × 10^−15^) among the top associations. Tissue-level expression analysis using GTEx project data revealed significant enrichment in skeletal muscle (*P* = 7.44 × 10^−7^) and heart atrial appendage (*P* = 6.46 × 10^−4^) (Fig. 2B; Supplementary Table S5). Single-cell transcriptomic data from the Human Protein Atlas further supported these findings, showing significant enrichment in skeletal myocytes (*P* = 4.39 × 10^−7^) and cardiomyocytes (*P* = 2.39 × 10^−4^) among 81 evaluated cell types, with monocytes ranking third (*P* = 3.23 × 10^−3^), though not significant after multiple testing correction (Fig. 2C; Supplementary Table S6). Pathway enrichment analysis highlighted Gene Ontology biological processes related to the negative regulation of muscle adaptation (*P* = 1.05 × 10^−6^), negative regulation of cardiac muscle adaptation (*P* = 1.59 × 10^−6^), and striated muscle cell development (*P* = 2.43 × 10^−6^) as the most significantly enriched (Supplementary Table S7).

### Functional landscape of CK-associated genetic loci

In the multi-ancestry GWAS meta-analysis comprising all cohorts, we identified 107 independent loci at genome-wide significance (*P*< 5 × 10^−8^), defined as regions within ±500 kb of each lead variant. Effect estimates are reported in Supplementary Table S8 to allow assessment of effect magnitude and direction. Of the 107 lead variants from the primary meta-analysis, heterogeneity metrics were available for 100 in the IVW meta-analysis excluding BBJ. Only seven of 100 lead variants showed nominal heterogeneity (P_het <0.05, Supplementary Table S8). Of the 107 loci, 98 loci represent previously unreported associations not previously reported in earlier serum CK GWAS.^4^^,^^5^^,^^21^ To assess their functional relevance, we annotated the 107 lead SNPs using publicly available resources (Supplementary Table S8), which informed downstream analyses.

Among the 107 loci, 15 lead variants mapped to exonic regions. The CK signals previously reported in *CKM* (*P* = 1.22 × 10^−219^), *LILRB5* (*P* = 1.11 × 10^−183^), and *ANO5* (*P* = 5.79 × 10^−60^) were replicated with the strongest associations in the multi-ancestry meta-analysis. All 15 exonic lead variants were missense, and ClinVar annotations indicate that 11 have previously been linked to Mendelian disorders, although mostly classified as benign. Notably, the newly identified rs3850625 (*P* = 4.89 × 10^−11^, p.R1539C) in *CACNA1S* has been implicated in congenital myopathy, malignant hyperthermia susceptibility, and periodic paralysis. Additional conditions associated with exonic variants include *MYPN*-related myopathy and dilated cardiomyopathy (for example, rs10997975 in *MYPN p.S691N*, *P* = 4.86 × 10^−29^), as well as disorders involving *TTN* (rs12463674, p.I17160T, *P* = 4.78 × 10^−22^), *MYO18B* (rs133885, p.G44E, *P* = 1.15 × 10^−11^), *NEB* (rs13013209, p.K2613N, *P* = 2.98 × 10^−16^) and *ANO5* (rs7481951, p.L321F, *P* = 5.79 × 10^−60^). Furthermore, an exonic variant in the non-coding RNA gene *TARID*, rs2277083 (*P* = 4.51 × 10^−18^), having prior ClinVar submissions referencing dilated cardiomyopathy 1J, was among the loci detected.

The majority of association signals arose from intronic regions, including 48 previously unreported loci (Supplementary Table S8). Several intronic lead variants were coincidential with muscle eQTL, implicating genes such as *STAT3* (rs3736161, *P* = 1.54 × 10^−13^), *SMAD3* (rs12901499, *P* = 3.20 × 10^−13^) and *CSF1* (rs333947, *P* = 1.22 × 10^−40^). Intergenic loci also occurred in the same region as muscle eQTL associations; for example, rs11714943 (*P* = 1.83 × 10^−12^), located between *PRKCI* and *SKIL*, were coincidental with an eQTL for *SKIL*. Similarly, the previously reported intronic variant rs9543398 (P = 2.91 × 10^−47^) in LINC00392 was coincidental with a muscle eQTL for *KLF5*.

Annotation against the GWAS Catalogue revealed extensive pleiotropy among loci associated with serum CK levels (Supplementary Table S8). These loci are frequently linked to traits related to tissue damage, lipid metabolism, body composition, and cardiovascular phenotypes. Exonic variants in genes with muscle-specific expression displayed particularly strong pleiotropic effects. For instance, rs12975366 (*P* = 1.11 × 10^−183^) in *LILRB5* is associated with high-density lipoprotein (HDL) cholesterol and leucocyte immunoglobulin-like receptor subfamily B member 5 levels, whereas rs10997975 in *MYPN* associates with hip circumference (adjusted for body mass index), height and serum creatinine concentration. Similarly, rs3850625 in *CACNA1S* shows associations with aspartate aminotransferase (AST) levels, serum creatinine and whole-body fat-free mass.

### Shared genetic architecture with Mendelian muscle disorders

ClinVar annotations indicated a potential link between loci associated with serum CK levels and genes implicated in Mendelian muscle disorders. To investigate this relationship, we performed an enrichment analysis using a curated set of muscle-related Mendelian disorder genes from the Online Mendelian Inheritance in Man (OMIM) database (Supplementary Table S9). Among the 173 genes in this set, 8 overlapped with the 68 genes mapped to lead variants identified in the multi-ancestry CK GWAS meta-analysis (Table 1). This overlap represented a significant enrichment of CK-associated genes among those implicated in Mendelian muscle disorders (Fisher's exact test, odds ratio [OR] = 13.99, 95% confidence interval [CI] = 5.68–29.96, *P* = 3.38 × 10^−7^). Five of the CK-associated loci contained nonsynonymous lead variants; all five are reported in ClinVar for relevant Mendelian conditions. In contrast, the three intronic lead variants lacked corresponding ClinVar annotations.

**Table 1:** Lead SNPs at CK-associated loci and their mapped genes with Mendelian muscle disease associations.

SNP	Chr	Pos	Ref	Alt	Freq	*P*	Func	Gene	OMIM disease
rs3850625	1	201,016,296	G	A	0.07	4.89 × 10^−11^	exonic	*CACNA1S*	Malignant hyperthermia susceptibility 5; Congenital myopathy 18 due to dihydropyridine receptor defect
rs13013209	2	152,500,449	C	G	0.33	2.98 × 10^−16^	exonic	*NEB*	Nemaline myopathy 2, autosomal recessive
rs12463674	2	179,432,185	A	G	0.16	4.78 × 10^−22^	exonic	*TTN*	Cardiomyopathy, dilated, 1G; Congenital myopathy 5 with cardiomyopathy; Cardiomyopathy, familial hypertrophic, 9; Muscular dystrophy, limb-girdle, autosomal recessive 10; Myopathy, myofibrillar, 9, with early respiratory failure
rs237870	3	8,779,066	T	C	0.28	8.84 × 10^−12^	intronic	*CAV3*	Myopathy, distal, Tateyama type; Cardiomyopathy, familial hypertrophic
rs10997975	10	69,933,921	G	A	0.40	4.86 × 10^−29^	exonic	*MYPN*	Cardiomyopathy, familial restrictive, 4; Cardiomyopathy, hypertrophic, 22; Cardiomyopathy, dilated, 1 KK; Congenital myopathy 24
rs72842207	10	121,433,675	C	T	0.17	1.43 × 10^−19^	intronic	*BAG3*	Cardiomyopathy, dilated, 1HH; Myopathy, myofibrillar, 6
rs7481951	11	22,271,870	A	T	0.34	5.79 × 10^−60^	exonic	*ANO5*	Muscular dystrophy, limb-girdle, autosomal recessive 12; Miyoshi muscular dystrophy 3
rs11081948	18	33,987,478	A	G	0.42	4.19 × 10^−9^	intronic	*FHOD3*	Cardiomyopathy, familial hypertrophic, 28

SNP: lead variant representing the CK-associated locus; Chr: chromosome; Pos: genomic position (GRCh37, base pairs); Ref/Alt: reference and alternate alleles; Freq: frequency of the alternate allele in the multi-ancestry meta-analysis; P: association P-value for CK; Func: functional annotation of the lead SNP; Gene: gene mapped to the lead SNP; OMIM Disease: Mendelian muscle disease(s) associated with the gene in the OMIM database.

To further validate these findings, we repeated the enrichment analysis using all genome-wide significant SNPs from the primary multi-ancestry meta-analysis (including BBJ), rather than only the lead variants, which mapped to 221 CK-associated genes. Of these, 15 overlapped with the Mendelian muscle gene set, again indicating significant enrichment (Fisher's exact test, OR = 7.91, 95% CI = 4.25–13.75, *P* = 5.18 × 10^−9^). The full list of genes and associated disorders is provided in Supplementary Table S10.

### Mendelian randomisation and colocalisation prioritise candidate genes

**Fig. 3:**
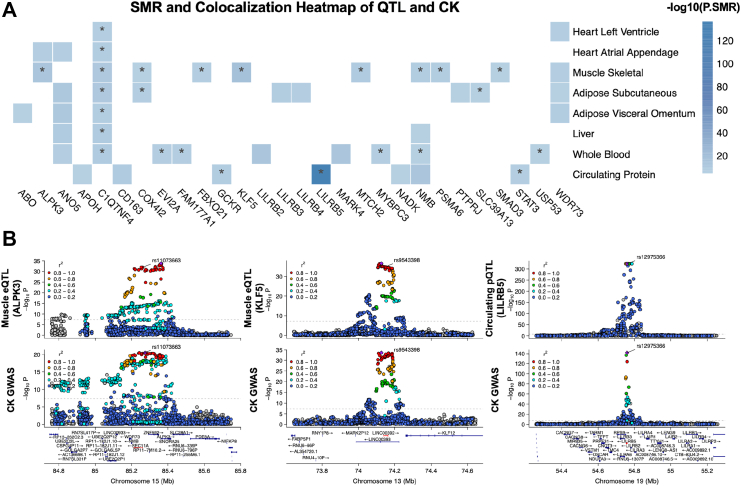
**Mendelian Randomisation and colocalisation analyses implicate candidate genes for serum CK.** (A) Heatmap of SMR associations (−log_10_P) between eQTLs and pQTLs across heart, skeletal muscle, adipose, liver, whole blood, and circulating proteins versus CK GWAS signals. Asterisks indicate significant colocalisation. (B) Regional plots for ALPK3 in muscle (rs11073663), KLF5 in muscle (rs9543398), and LILRB5 in plasma proteins (rs12975366), showing QTL signals above and CK GWAS signals below. Variants are coloured by LD r^2^ with the lead SNP.

Functional annotation highlighted that multiple CK lead SNPs were coincidental with eQTLs across various tissues, particularly in muscle. We performed SMR and colocalisation analyses, which further highlighted candidate genes potentially involved in muscle-related determinants of CK leakage/clearance. The heat map in Fig. 3A summarises the QTL-based SMR and colocalisation results. After correction for multiple testing ($P_{SMR}$< 1.70 × 10^−6^), we identified 42 significant SMR associations, predominantly in skeletal muscle, whole blood and subcutaneous adipose tissue. Of these, 23 associations passed the heterogeneity in dependent instruments (HEIDI) test ($P_{HEIDI}$> 0.05), indicating consistency with vertical pleiotropy (Supplementary Table S11). Notably, the *LILRB5* pQTL emerged as the most significant association ($P_{SMR}$ = 3.11 × 10^−137^, $\beta_{SMR}$ = 0.104, ${SE}_{SMR}$ = 0.004). Additionally, *KLF5* ($P_{SMR}$ = 5.23 × 10^−19^, $\beta_{SMR}$ = −0.155, ${SE}_{SMR}$ = 0.017), *SMAD3* ($P_{SMR}$ = 1.14 × 10^−6^, $\beta_{SMR}$ = −0.108, ${SE}_{SMR}$ = 0.022) and *ALPK3* ($P_{SMR}$ = 1.81 × 10^−13^, $\beta_{SMR}$ = −0.160, ${SE}_{SMR}$ = 0.022) appeared exclusively in muscle-specific SMR analyses. Several genes were implicated across multiple tissues, including *ANO5*, which showed consistent associations in whole blood, liver, heart and adipose tissue, but not in skeletal muscle. The expression of *C1QTNF4* was associated with CK in all seven tissues analysed.

Colocalisation analyses provided further support for the SMR findings, particularly in skeletal muscle (Fig. 3B and Supplementary Table S12). Genes such as *ALPK3* (posterior probability for colocalisation [${\text{PP}}_{H4}$] = 0.996), *SMAD3* (${\text{PP}}_{H4}$ = 0.917) and *KLF5* (${\text{PP}}_{H4}$ = 0.987) demonstrated high posterior probabilities for a shared causal variant influencing both gene expression in muscle and CK levels. Conversely, *LILRB2* exhibited a strong SMR signal in whole blood ($P_{SMR}$ = 9.86 × 10^−14^, $\beta_{SMR}$ = 0.473, ${SE}_{SMR}$ = 0.064) but lacked colocalisation support (${\text{PP}}_{H4}$ = 1.92 × 10^−4^). The circulating GCKR (${\text{PP}}_{H4}$ = 0.977) and LILRB5 (${\text{PP}}_{H4}$ = 1.000) pQTLs were further supported by strong colocalisation evidence (Supplementary Table S12). Representative colocalisation plots for the eQTL of *ALPK3* and *KLF5* in muscle, as well as the pQTL of LILRB5, are shown in Fig. 3B.

To further contextualise genes prioritised by skeletal muscle eQTL colocalisation, we interrogated MetaMEx for meta-analysed skeletal muscle expression changes following exercise and inactivity (Supplementary Figure S6). *ALPK3*, *KLF5*, and *SMAD3* showed the clearest acute exercise–responsive signals, with *ALPK3* and *KLF5* significantly upregulated after acute resistance exercise and *SMAD3* significantly upregulated after acute aerobic exercise (adjusted *P*< 0.05), with generally concordant directions across acute exercise modalities. In contrast, other colocalized genes showed limited evidence of differential expression across the evaluated interventions.

### Pleiotropic effects of CK-associated genetic loci

GWAS Catalogue annotations indicated extensive pleiotropy among CK-associated loci, spanning biomarkers, haematological traits, muscle phenotypes, and disease outcomes, partially mirroring genome-wide genetic correlations. On this basis, we assembled a set of CK-related traits, including quantitative biomarkers, muscle phenotypes, and diseases for correlation and pleiotropy analyses.

**Fig. 4:**
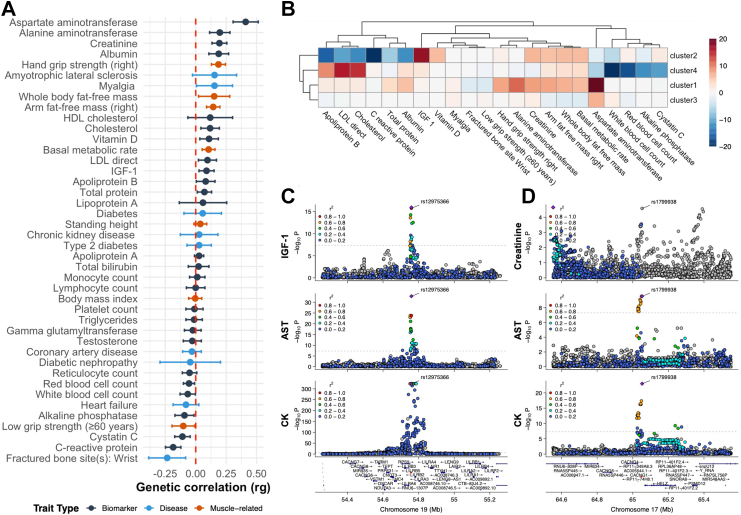
**Genetic correlations and pleiotropic effects of CK-associated loci.** (A) Genome-wide genetic correlations between CK and 42 systemic traits estimated by LDSC. Traits are grouped as biomarkers (black), diseases (blue), and muscle-related traits (red). Horizontal bars denote 95% CI; red dashed line, zero correlation. (B) Heatmap summarising pleiotropic patterns across clusters. Each cell shows signed −log_10_(P) for the association between cluster membership and the trait (sign from the regression coefficient aligned to the CK-increasing allele). Values are truncated at ±20 for visualisation; positive indicates trait-increasing, negative indicates trait-decreasing effects. (C) Regional plot for rs12975366 showing colocalisation of IGF-1, AST, and CK signals. Dotted line, genome-wide significance (P = 5 × 10^−8^); points coloured by LD r^2^ with lead SNP. (D) Regional plot for rs1799938 demonstrating shared effects on creatinine, AST, and CK.

Genetic correlations between serum CK levels and related traits were evaluated using LDSC in the European ancestry cohorts (Fig. 4A). Full results are provided in Supplementary Tables S13 and S14. Among 42 systemic traits, nine showed significant genetic correlations after Bonferroni correction (*P* < 1.19 × 10^−3^). The strongest positive correlation was observed with AST levels ($r_{g}\;\left(LDSC\right)$ = 0.410, *P* = 6.88 × 10^−15^), followed by muscle-related traits, including hand grip strength ($r_{g}$ = 0.187, *P* = 2.35 × 10^−10^), arm fat-free mass ($r_{g}$ = 0.144, *P* = 2.87 × 10^−7^), and basal metabolic rate ($r_{g}$ = 0.106, *P* = 8.14 × 10^−5^). Similarly, in analyses of cardiorespiratory function and cardiac structural traits, significant correlations were primarily observed for fitness- and activity-related phenotypes, including cardiorespiratory fitness (${VO}_{2}\;\max$; $r_{g}$ = 0.259, *P* = 6.94 × 10^−5^) and physical activity ($r_{g}$ = 0.202, *P* = 1.02 × 10^−6^), as well as for right ventricular stroke volume ($r_{g}$ = 0.191, *P* = 0.001). Other imaging-derived ventricular measures and cardiorespiratory disease traits showed no significant genetic correlation after Bonferroni correction (Supplementary Table S14). CK levels were also significantly correlated with multiple circulating biomarkers, including alanine aminotransferase (ALT; $r_{g}$ = 0.198, *P* = 3.60 × 10^−6^), creatinine ($r_{g}$ = 0.195, *P* = 3.87 × 10^−10^), albumin ($r_{g}$ = 0.189, *P* = 5.17 × 10^−6^), and cystatin C ($r_{g}$ = −0.109, *P* = 8.00 × 10^−4^), as well as the inflammatory marker CRP ($r_{g}$ = −0.185, *P* = 1.10 × 10^−8^) (Supplementary Table S13).

To explore potential causal relationships underlying the systemic trait associations with CK, we conducted two-sample MR analyses for traits used in the genetic correlation analysis (Supplementary Table S15). Significant causal effects ($P_{IVW}$ < 6.10 × 10^−4^) on CK were observed for AST ($P_{IVW}$ = 7.74 × 10^−9^, $\beta_{IVW}$ = 0.274), arm fat-free mass ($P_{IVW}$ = 5.15 × 10^−9^, $\beta_{IVW}$ = 0.170), hand grip strength ($P_{IVW}$ = 7.91 × 10^−5^, $\beta_{IVW}$ = 0.246), and creatinine ($P_{IVW}$ = 1.76 × 10^−4^, $\beta_{IVW}$ = 0.099). Conversely, when CK was used as the exposure, only AST showed significant evidence of a causal effect ($P_{IVW}$ = 1.17 × 10^−4^, $\beta_{IVW}$ = 0.164). Instrumental variable plots and MR estimates for AST, hand grip strength, and arm fat-free mass are shown in Supplementary Figure S7. All traits demonstrated substantial heterogeneity across instrumental variables (Cochran's Q test, *P* < 0.05), and Egger's test indicated potential horizontal pleiotropy for AST both as exposure ($P_{Egger}$ = 0.001) and outcome ($P_{Egger}$ = 0.014).

Effect sizes of lead SNPs across correlated systemic traits further informed the functional characterisation of CK-associated loci through clustering and multi-trait colocalisation analyses. Eight lead SNPs lacking coverage across most traits and 20 traits with weak genetic correlations with CK ($P_{LDSC}$> 0.05) were excluded. The remaining 99 lead variants were grouped into four clusters based on their effect sizes across 22 remaining traits (Supplementary Figure S8 and Supplementary Table S16). Fig. 4b summarises cluster–trait associations as signed −log_10_(P) values aligned to the CK-increasing allele. Notably, Clusters 1 and 3 accounted for the majority of lead variants (95 of 99). These two clusters showed consistent positive associations with AST (*P* = 8.76 × 10^−24^ for Cluster 1; *P* = 7.53 × 10^−8^ for Cluster 3), with Cluster 1 also exhibiting associations with ALT (*P* = 4.50 × 10^−12^) and creatinine (Fig. 4B). By contrast, Clusters 2 and 4 exhibited negative associations with AST levels and white blood cell count (Fig. 4B). Cluster 2 was also negatively associated with cholesterol and low-density lipoprotein (LDL) levels, whereas Cluster 4 showed positive associations with these lipid traits. Cluster 2 includes rs1260326, a coding variant in *GCKR*. Cluster 4 comprises loci mapping to *SH2B3*, *PTPN11*, and *ABO*.

Multi-trait colocalisation analysis confirmed shared genetic signals between CK and these traits (Supplementary Table S17), particularly AST and ALT. For example, the CK-associated locus rs7481951 in *ANO5* (posterior probability [PP] = 0.999) colocalized with both AST and ALT. The rs12975366 variant in *LILRB5* colocalized with AST and insulin-like growth factor 1 (IGF-1) (PP = 0.959), while rs1799938 in *CACNG1* colocalized with AST (PP = 0.835). Cross-trait regional plots of *LILRB5* and *CACNG1* are shown in Fig. 4C and D. However, colocalisation of CK signals in cluster 2 and 4 with other traits could not be confirmed.

## Discussion

Creatine kinase plays a central role in muscle energy metabolism and serves as a widely used clinical biomarker of muscle damage. Despite its clinical utility, serum CK levels exhibit considerable inter-individual variability, limiting the ability to differentiate between normal physiological fluctuations and pathological elevations.^3^ To elucidate the genetic architecture underlying CK variation, we performed a large multi-ancestry GWAS meta-analysis of serum CK levels.

This study examines variation in serum CK levels within population biobanks, recognising that genetic associations may differ in disease-specific contexts. Most samples were drawn from the BBJ and MVP cohorts. Although BBJ includes individuals with diverse clinical conditions, disease status was included as a covariate in CK GWAS, and the maximum CK value of 332 units per litre (U/L) suggests that extreme elevations associated with acute damage were likely absent. In MVP, individuals with markedly elevated CK, typically reflecting acute muscle damage, were excluded. These measures help to minimise confounding from disease-related elevations in CK. Additionally, a rank-based inverse normal transformation was applied in MVP, QGP, and VUMC cohorts to reduce the influence of outliers. These steps are expected to reduce the influence of acute or extreme elevations and limit confounding from overt muscle injury; however, residual effects from transient elevations and unmeasured clinical context cannot be fully excluded in biobank-based CK measurements.

Ancestry-specific effect sizes of significant SNPs were moderately concordant in direction and magnitude across six cohorts and were combined in multi-ancestry meta-analysis. Although nominally significant heterogeneity was observed at 7 loci, the overall extent of heterogeneity appeared limited, particularly given the multi-ancestry framework of the analysis. SNP-based heritability, estimated using LDSC, was consistent across the MVP European ancestry subset, VUMC and BBJ, yielding an overall estimate of approximately 10%. This estimate is similar to SNP-heritability reported for several circulating biomarkers.^11^ These results establish a conceptual basis for evaluating the clinical implications of genetically determined variation in CK. High-sensitivity C-reactive protein (hsCRP) provides a precedent: in large population-based cohorts, it independently predicts major adverse cardiovascular events and improves risk discrimination in individuals without established atherosclerotic cardiovascular disease, supporting its use in primary prevention.^40^ Constitutional differences in CK driven by germline variation may prove informative in selected clinical or physiological contexts. Prospective studies could evaluate whether polygenic risk score (PRS) derived from CK-associated variants provide additional information in contexts in which muscle injury is clinically relevant, such as statin exposure or susceptibility to exercise-induced muscle damage. Given the biological heterogeneity of CK-associated loci, stratified or pathway-informed PRSs may prove more informative than a single aggregated score.

MAGMA analyses revealed genome-wide enrichment across tissues, cell types, and pathways, with recurrent signals in muscle-related contexts. Pronounced muscle specificity was also observed for loci mapping to key muscle-function genes, including *ANO5*, *TTN*, and *MYPN*, with eQTL colocalisation signals predominantly observed in muscle tissue compared to other tissues. Together, these findings are consistent with the interpretation that the genetic regulation of serum CK variation in biobank settings partly reflects intrinsic muscle homoeostasis. Although not statistically significant, enrichment in monocytes and granulocytes, ranking third and fourth, was comparable with previous GWAS findings in an Icelandic cohort and supports a potential role for monocyte/macrophage-mediated CK clearance,^4^ in line with associations at *CSF1*.

We assessed genome-wide genetic correlations between serum CK and a broad range of physiological and disease-related traits. The strongest positive correlations were observed with liver enzymes, particularly AST, likely reflecting shared tissue origin of skeletal muscle and biology of enzyme release.^41^ The colocalisation of loci in muscle-specific genes, such as *CACNG1* and *ANO5*, suggests that the elevated AST levels originate from muscle release. Trait-based clustering further supported this link, with CK-associated loci generally aligning in effect direction with AST. In contrast, ALT showed a weaker correlation, likely due to its liver-specific expression.^42^ CK also showed a positive genetic correlation with creatinine, a metabolite derived from muscle.^43^ Among disease traits, nominal positive correlations were observed with amyotrophic lateral sclerosis and myalgia, though these did not reach statistical significance. Notably, CK was negatively correlated with wrist fracture, possibly reflecting underlying differences in muscle strength and reduced fracture risk.^44^ This was supported by positive correlations between CK and both hand grip strength and fat-free mass, highlighting the influence of muscle mass and physical activity on serum CK levels.^45^ Consistent with these activity- and muscle-related correlations, we additionally observed positive genetic correlations with cardiorespiratory fitness (VO2max) and physical activity, as well as right ventricular stroke volume. CK was negatively correlated with inflammatory markers such as CRP and leucocyte count, showing agreement with reports linking lower CK levels to haematologic malignancies and increased immune-mediated enzyme clearance.^46^^,^^47^ These findings imply that heightened immune activity may reduce CK levels through enhanced clearance mechanisms. Positive correlations were also observed with total cholesterol and LDL, suggesting an interaction between muscle and lipid metabolism. The significant correlation with albumin and cystatin C lacks strong support in current literature and warrants further study using individual-level data. We note that these genome-wide correlations reflect average, intricate patterns of CK and related traits at the population level. However, locus-specific effects may vary in direction, as the two-sample MR analyses showed extensive instrumental variable heterogeneity and horizontal pleiotropy. The causality among these traits may need to be evaluated at the variant level and may differ across loci.

The multi-ancestry meta-analysis identified 107 loci associated with CK levels, including 98 not previously reported. Here, we focus on loci with strong prior links to muscle biology, exonic variants implicated in Mendelian disorders, and loci exhibiting distinct pleiotropic patterns across trait-defined clusters; additional loci are described in the Supplementary Note. Notably, multiple loci carrying exonic nonsynonymous variants were mapped to genes essential for muscle structure and sarcolemmal integrity. The genes in these CK loci were significantly enriched in a Mendelian muscle disorder gene set, which is consistent with reports from other complex traits.^48^ This enrichment provides orthogonal support that the genetic determinants of serum CK variation are not randomly distributed across the genome but are preferentially connected to genes with established relevance to inherited muscle pathology. For example, the variant rs12463674 lies within a conserved A-band genomic region of the *TTN* gene, a known hotspot for titinopathy mutations.^49^ Variants in *NEB* (rs13013209) and *MYPN* (rs10997975), core Z-line and sarcomere components, are associated with CK efflux and are annotated in ClinVar for benign myopathy.^50^^,^^51^ A previously unreported association at rs3850625 in *CACNA1S*, encoding the α1S subunit of the skeletal muscle L-type calcium channel, colocalizes between CK levels, grip strength, and lean mass, supporting a shared role in excitation–contraction coupling.^52^ ClinVar links this variant to benign congenital myopathy and malignant hyperthermia susceptibility.^52^^,^^53^ Similar associations were observed at rs1799938 in *CACNG1* and rs7481951 in *ANO5*, both involved in calcium homoeostasis and membrane repair.^54^^,^^55^ *ANO5* has been previously linked to muscular dystrophies and variation in CK levels.^4^^,^^7^ Notably, additional signals were mapped to non-coding regions of ion channel genes, including *KCNMA1* and *KCNJ2*, further underscoring the importance of ion regulation. In addition to five OMIM-reported genes—*CACNA1S*, *MYPN*, *ANO5*, *NEB*, and *TTN*—numerous other loci were associated with skeletal and cardiac muscle phenotypes, although many remain functionally uncharacterised. An association was identified at rs133885 in *MYO18B*, involved in sarcomere assembly in fast skeletal muscle^56^; although associated with neurodevelopmental syndromes such as Klippel–Feil anomaly,^57^ its role in muscle remains poorly defined. Lastly, rs10857472, a nonsynonymous variant in *C10orf71*, lies in a gene where frameshift mutations have been linked to dilated cardiomyopathy in humans, mice, and cardiac organoids.^58^

Although the identified exonic variants are common and likely not fully penetrant, their presence in genes linked to Mendelian myopathies suggests they may act as genetic modifiers or influence disease susceptibility under certain conditions. Variants in *TTN*, *NEB*, and *ANO5*—genes associated with recessive muscular disorders—could subtly disrupt sarcolemmal stability or calcium homoeostasis.^55^^,^^59^ These subclinical effects may increase vulnerability to muscle injury–related CK elevations in response to stressors like intense exercise, infection, or medication. While the lack of individual-level and longitudinal data limits direct risk assessment, the involvement of these variants in structural and signalling pathways essential to muscle function supports a model in which common alleles influence muscle resilience. Elevated serum CK levels in genetically predisposed individuals may reflect subtle differences in sarcolemmal integrity, unmasked by additional genetic or environmental factors. This model is consistent with clinical observations, where subclinical muscle disorders have emerged following exposure to agents such as statins. In one study of 110 individuals with drug-induced myopathies, 10% carried heterozygous or homozygous pathogenic mutations in genes known to cause these disorders,^60^ highlighting the value of genetic testing even in mild or unexplained cases.

Candidate gene prioritisation identified putative mediators of CK variation and muscle biology, particularly at non-coding loci. In addition to genes implicated in muscle function (*ALPK3*) and immune regulation (*LILRB5*), components of the transforming growth factor-beta (TGF-β) signalling pathway were recurrently highlighted, with several loci mapping to its regulatory genes. Among these, the CK signal at an intronic variant in *SMAD3* (rs12901499) colocalizes with its muscle eQTL, and eQTL-based MR supports a causal link between increased *SMAD3* expression and reduced CK levels. *SMAD3*, a central mediator of TGF-β signalling, is essential for muscle mass maintenance and satellite cell function.^61^ Its loss leads to atrophy, impaired myoblast differentiation, and elevated myostatin expression, a known inhibitor of muscle growth.^61^^,^^62^ Two additional loci further implicate TGF-β signalling. At *SKIL* (SnoN), muscle eQTLs were detected, although without colocalisation with CK association signals. Given *SKIL*'s role as a SMAD-recruited transcriptional repressor,^63^ and its degradation upon *SMAD3* activation, variation at this locus may modulate pathway output, pending functional validation. At *STAT3*, a key node in cytokine–TGF-β crosstalk,^64^ colocalisation and pQTL-MR suggest that genetically elevated circulating STAT3 is associated with lower CK levels. A locus near LINC00392, close to the 13q22.1 signal reported in an Icelandic CK study,^4^ colocalizes with *KLF5*, whose increased expression in muscle is causally linked to reduced CK. *KLF5* is involved in exercise-induced lipid remodelling—promoting lipid oxidation acutely, and lipid synthesis with chronic training—and contributes to muscle atrophy through coordination with *FOXO1* and E3 ubiquitin ligases.^65^^,^^66^ Notably, MetaMEx analyses showed that *ALPK3*, *KLF5*, and *SMAD3* exhibit significant acute exercise–responsive expression changes in skeletal muscle, consistent with roles in muscle adaptation programs. However, it remains unclear whether the acute, exercise-induced transcriptional responses observed in MetaMEx reflect the same expression-mediated mechanism captured by CK GWAS-muscle eQTL colocalisation or instead indicate broader roles for these genes in muscle adaptation independent of CK-associated regulatory effects. KLF5 also engages in bidirectional crosstalk with the TGF-β pathway: it promotes TGF-β expression and profibrotic signalling during fibroblast-driven remodelling, while active SMAD2/3–SMAD4 complexes recruit p300 to acetylate *KLF5*, forming a KLF5–SMAD–p300 complex that drives TGF-β target gene expression.^67^ This positions *KLF5* as a potential integrator of TGF-β signalling with metabolic and proteolytic muscle responses, linking CK genetics to the broader TGF-β/SMAD axis involving *SMAD3*, *STAT3*, and *SKIL*. Other TGF-β–related genes, including *SMAD7* and *BACH1*, showed no eQTL colocalisation, leaving their roles in CK regulation unresolved. Together, these findings highlight that these genetic variants may influence CK regulation and muscle-related pathways through TGF-β signalling.

Analysis of pleiotropic effects revealed that most loci are primarily associated with a predominantly muscle-linked biomarker profile, as indicated by consistent effects on tissue damage biomarkers such as AST at the *CACNG1* and *LILRB5* loci. This pattern aligns with the established roles of genes at these loci in maintaining muscle structural integrity and contraction function. In contrast, four loci identified through trait-based clustering exhibited divergent pleiotropic signatures, indicative of more complex underlying biological processes. The variant rs1260326 in an exon of *GCKR* defines Cluster 2 and exhibits a concordant association with serum levels of CK and creatinine, but a discordant association with AST. This locus is also linked to systemic traits such as CRP and body mass index. *GCKR*, a key regulator of hepatic and pancreatic glucokinase, plays a central role in glucose metabolism and is a well-established locus for metabolic traits and disorders.^68^ The genetic association with CK may reflect pleiotropic effects mediated through multiple metabolic pathways. Colocalisation and the positive effect of circulating *GCKR* levels on CK suggest this locus may be a viable therapeutic target. The loci *SH2B3*, *PTPN11*, and *ABO* form Cluster 4, characterised by associations with both immune and metabolic traits. *SH2B3* is a negative regulator of cytokine signalling essential for haematopoiesis,^69^ while *ABO* is best known for determining blood group. *PTPN11* encodes a tyrosine phosphatase involved in key signalling pathways and is associated with Noonan syndrome and acute myeloid leukaemia.^70^ This cluster likely captures haematopoietic effects, consistent with observed reductions in CK in haematologic malignancies.^46^ Outside of Cluster 4, the LINC02227/EBF1 locus also demonstrates haematopoietic relevance, showing colocalisation with lymphocyte count. *EBF1* has been implicated in immune-related diseases such as acute lymphoblastic leukaemia.^71^

While our findings reflect the genetic architecture of population CK levels, we could not fully exclude individuals with acute conditions, and some residual influence from transient elevations may remain. Key time-varying determinants, most notably physical activity and recent exercise, as well as intercurrent injury or infection and medication exposures, were not comprehensively captured, leaving scope for residual confounding. Physical activity is particularly challenging to handle because it may function as both a confounder and a mediator of genetic effects on muscle physiology and CK. Moreover, the lack of individual-level data limits deeper analyses of CK distributions, genotype-specific risk, and covariate-adjusted models. It also limits stratified or interaction inference across activity levels and disease subtypes, which warrant future investigation. Finally, independent replication in additional well-powered multi-ancestry cohorts is important to confirm the robustness and generalisability of the newly identified loci.

In summary, we present a large multi-ancestry meta-analysis of serum CK levels, identifying previously unreported genetic loci and providing preliminary insights into their potential roles in serum CK variation and underlying muscle-related biology. These results refine understanding of constitutional CK differences in population settings and motivate future work evaluating genetic scores and variants in clinically relevant contexts.

## Contributors

Conceptualisation: SSZ, APM, JAL, GC; Data curation: GC and SSZ curated and verified the GWAS summary statistics used in this study; GC and JBL curated and verified the OMIM-derived Mendelian muscle disease gene set; Methodology: APM, JAL, GC, SSZ, WL; Formal analysis: GC, WL (pQTL MR); Visualisation: GC; Supervision: APM, JAL, HC; Writing—original draft: GC; Writing—review & editing: all authors. No individual-level data were accessed in this study. These authors contributed equally: APM, JAL. All authors read and approved the final manuscript.

## Data sharing statement

The GWAS summary statistics generated in this study will be publicly available through the GWAS Catalogue under accession number GCST90832189 upon publication. This study used de-identified summary-level data only, and no individual-level participant data were accessed or generated. Additional summary results supporting the findings of this study are available from the authors upon reasonable request.

Correspondence and requests for materials should be addressed to APM or JAL.

## Declaration of interests

SSZ is supported by a Versus Arthritis Career Development Fellowship (grant no. 23258), and works in centres supported by Versus Arthritis (grant no. 21173, 21754 and 21755) and the NIHR Manchester Biomedical Research Centre (NIHR203308). WL is supported by the Guangzhou Elite Project (project no. JY202314). YHL is supported by the China Scholarship Council (202406010039). APM is supported by Versus Arthritis (21574), NIHR Manchester Biomedical Research Centre (NIHR203308), and Medical Research Council (MR/W029626/1). JAL is supported by Myositis UK (007_SG_MyoUK, 014_LG_MyoUK), Cancer Research UK, and collaborative research funding from Pfizer Limited. This study has been delivered through the National Institute for Health and Care Research (NIHR) Manchester Biomedical Research Centre (NIHR203308). The views expressed are those of the author(s) and not necessarily those of the NIHR or the Department of Health and Social Care.

The authors declare that they have no competing interests.
